# Glycerine

**Published:** 1849-11

**Authors:** 


*Glycerine.*—Mr. Burnett, Tremont Row, has this new and elegant article, which is beginning to have a reputation with the profession for the amelioration and cure of various cutaneous eruptions. Without attempting to describe the process of making glycerine, it is proper to observe that in soap-making, in some common method familiar to pharmaceutists, this oily, almost inodorous substance is obtained—vulgarly known, in olden times, as oil of lead. Dr. Durkee, of Howard Street, a gentleman devoted to the management of diseases of the skin, and whose success is acknowledged by the community, speaks very favorably of it, we understand. This suggestion, we trust, will have the effect of directing the attention of physicians to a preparation that may be serviceable, where others have failed.—*Boston Med. & Surg. Journal.*

